# Deep convolutional neural network VGG-16 model for differential diagnosing of papillary thyroid carcinomas in cytological images: a pilot study

**DOI:** 10.7150/jca.28769

**Published:** 2019-08-27

**Authors:** Qing Guan, Yunjun Wang, Bo Ping, Duanshu Li, Jiajun Du, Yu Qin, Hongtao Lu, Xiaochun Wan, Jun Xiang

**Affiliations:** 1 Department of Head and Neck Surgery, Fudan University Shanghai Cancer Center, Shanghai, 200032, China; 2 Department of Oncology, Shanghai Medical Colloge, Fudan University, Shanghai, 200032, China; 3 Depertment of Computer Science and Engineering, Shanghai Jiaotong University, Shanghai, China; 4 Department of Pathology, Fudan University Shanghai Cancer Center, Shanghai, 200032, China; Qing Guan and Yunjun Wang contributed equally to the work and should be regarded as co-first authors.

**Keywords:** Deep convolutional neural network, papillary thyroid carcinoma, cytological images, fine-needle aspiration, liquid-based cytology

## Abstract

**Objective**: In this study, we exploited a VGG-16 deep convolutional neural network (DCNN) model to differentiate papillary thyroid carcinoma (PTC) from benign thyroid nodules using cytological images.

**Methods**: A pathology-proven dataset was built from 279 cytological images of thyroid nodules. The images were cropped into fragmented images and divided into a training dataset and a test dataset. VGG-16 and Inception-v3 DCNNs were trained and tested to make differential diagnoses. The characteristics of tumor cell nucleus were quantified as contours, perimeter, area and mean of pixel intensity and compared using independent Student's t-tests.

**Results**: In the test group, the accuracy rates of the VGG-16 model and Inception-v3 on fragmented images were 97.66% and 92.75%, respectively, and the accuracy rates of VGG-16 and Inception-v3 in patients were 95% and 87.5%, respectively. The contours, perimeter, area and mean of pixel intensity of PTC in fragmented images were more than the benign nodules, which were 61.01±17.10 vs 47.00±24.08, p=0.000, 134.99±21.42 vs 62.40±29.15, p=0.000, 1770.89±627.22 vs 1157.27±722.23, p=0.013, 165.84±26.33 vs 132.94±28.73, p=0.000), respectively.

**Conclusion**: In summary, after training with a large dataset, the DCNN VGG-16 model showed great potential in facilitating PTC diagnosis from cytological images. The contours, perimeter, area and mean of pixel intensity of PTC in fragmented images were more than the benign nodules.

## Introduction

Thyroid nodules are a common medical condition, and most occurrences result in a benign outcome 1. However, differential diagnosis of thyroid nodules is crucial because thyroid carcinoma requires surgery, but only follow-up care is required for benign nodules. An accurate evaluation of thyroid nodules is suggested by the American Thyroid Association (ATA) guidelines along with recommendations about neck ultrasonography and fine-needle aspiration (FNA) cytology 2. Cytological evaluation of an FNA biopsy specimen remains the most precise single test for evaluating thyroid nodules to detect potential carcinomas. Thyroid carcinoma has four major pathology types: papillary thyroid carcinoma, follicular thyroid carcinoma (FTC), medullary thyroid carcinoma (MTC) and anaplastic thyroid carcinoma (ATC). Among these, PTC is the most frequently diagnosed type 3. The most common benign thyroid lesion is nodular goiter. Because FTC requires histology for definitive diagnosis and the incidences of MTC and ATC are relatively low, most studies concerning thyroid cytology choose to concentrate on differentiating PTC from benign nodules.

Applications that use machine learning (ML) have been increasing rapidly in the medical imaging field and have been applied to pathological diagnoses of various diseases 4-5. The DCNN is a type of ML constructed using a special type of artificial neural network that resembles the multilayered human cognition system. Many research groups have investigated applications of DCNNs to pathological images 6-9. Korbar et al. developed a system that used a DCNN to classify different types of colorectal polyps on whole-slide images 10. Ertosun et al. proposed an automated system to grade gliomas using DCNN 11. Most recently, an automated DCNN scheme was developed to classify lung carcinomas using cytological images. This system achieved excellent performance and ensured a promising role for DCNNs in cytological diagnosis 12.

Several studies have developed computer-assisted diagnostic systems for thyroid nodule cytology using models such as the Elman neural network (ENN) or the support vector machine (SVM) 13-15. However, to our knowledge, a DCNN has not previously been applied to diagnose cytological images of thyroid nodules. In this study, we retrained two automated DCNN schemes to classify thyroid nodules with cytological images, which were VGG-16^16^ and Inception-V3^17^. VGG-16 is a convolutional neural network model proposed by K. Simonyan and A. Zisserman from the University of Oxford, this model achieved 92.7% top-5 test accuracy in ImageNet, which is a dataset of over 14 million images belonging to 1000 classes^16^. Inception-v3 is also a convolutional neural network that is trained on more than a million images from the ImageNet database. The training results of these two models were compared at the end of this study.

## Materials and Methods

### Patients and cytological images

This study was conducted with the approval of the ethics committee of Fudan University Shanghai Cancer Center (FUSCC). The methods were conducted in accordance with the approved guidelines. The cytological images required to develop and evaluate our method were collected from patients who underwent thyroid nodule FNA and thyroidectomy from January 1^st^, 2017 to July 31^st^, 2017. Before the FNA procedure, written informed consent was obtained from all patients. An FNA biopsy was performed with 22-gauge needles by an experienced sonographer under ultrasonographic guidance. Thin layer liquid-based cytology (LBC) preparations are superior to conventional preparations with regard to background clarity, monolayer cell preparation and cell preservation. It is easier and less time consuming to screen and interpret LBC preparations because the cells are limited to smaller areas on clear backgrounds with excellent cellular preservation. Therefore, most of the FNA samples were transferred to a 10 ml syringe and then prepared with a natural sedimentation-type thin layer LBC system using a BD SurePath liquid-based Pap Test (Beckton Dickinson, Durham, NC, USA). Moreover, because most cytological pathologists in China were more accustomed to hematoxylin-eosin (H&E) staining, which can be easily compared with histological cell morphology, the LBC smears were usually processed with H&E staining. A digital still camera (DP27, Olympus, Tokyo, Japan) with a 40× objective lens attached to a microscope (BX45, Olympus) was used to take the pictures for the LBC smears. All photos were collected by experienced cytopathologists and saved in JEPG format.

**Figure 1:**
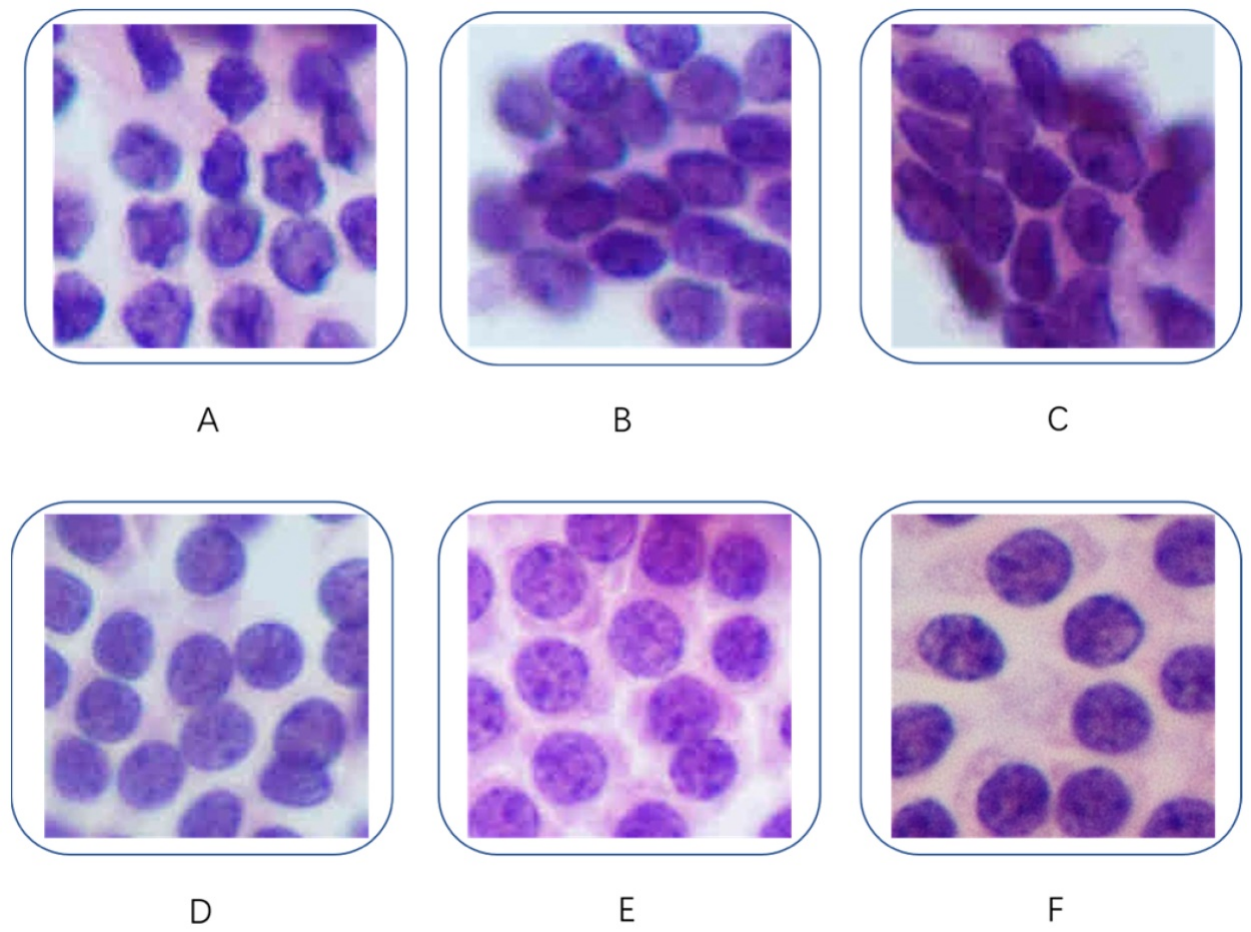
Fragmented cytological images. (A, B, C): PTC; (D, E, F): benign nodules.

Our dataset contained 279 H&E-stained images, each of which was associated with a different patient. The dataset included 159 cases of PTC and 120 cases of benign lesions. These slides were digitized at 400× magnification. Figure 1 shows some examples of the H&E-stained PTC and benign thyroid nodule images used in this project. All the PTC images had classic features (including high cellularity, papillary fronds with anatomical edges, enlarged oval nuclei with longitudinal intranuclear grooves, nuclear crowding and overlapping, cellular swirls, and chewing gum colloid 18) and were defined by our experienced cytopathologists as class V or VI according to the Bethesda system for reporting thyroid cytopathology 19. All the selected patients underwent a thyroidectomy and were given a pathologic diagnosis of PTC. All the images of benign nodules fit the class II description in the Bethesda system and consisted of an adequately cellular specimen composed of varying proportions of colloid and benign follicular cells arranged as macrofollicles and macrofollicle fragments 19. The patients with benign nodules did not receive surgery; thus, their histological data were not available. Consequently, the ground truth was derived from other clinical, laboratory, and imaging evaluations by an expert.

### Data augmentation

In the dataset, each image is manually segmented into several 224×224 fragments that contain several cells. As shown in Table 1, this segmentation resulted in a total of 887 224×224 fragmented images representing 476 PTC and 411 benign nodules. We split the dataset randomly into a training subset and a test subset for each cytology type at a ratio of approximately 6:1. We obtained 759 fragmented images for training and 128 fragmented images for testing with no overlap from the original images, resulting in 136 PTC and 103 benign nodule images in the training group and 23 PTC and 17 benign nodule whole-slide images in the test group.

We augmented the training data by flipping and rotating the images. Each image fragment was flipped horizontally and rotated by 0º, 90º, 180º and 270º. Through this flipping and rotating process, we increased the size of the training data by 8 times. If we were instead to directly augment the training data, the required storage space would expand by 8 times. Thus, to save storage space, we do not augment the training data in advance but only during the training process. In one iteration of the training process, we fetch a batch of images from the training data. We flip and rotate each image in the batch randomly. For each image, we randomly apply only one of the 8 transformation choices.

### Quantification of tumor cells

In order to obtain the characteristics of the tumor cells, we decided to find the contour features in the image to quantify the characteristics of tumor cell nucleus. As shown in Figure 3, we first converted the tumor images to grayscale images (Figure 3B), then we used the Laplacian method to calculate the second partial derivative of the image pixels in order to obtain contours in each tumor image (Figure 3C). After we extracted all the cell contours in each tumor image, then we counted the number of contours in each image, and we calculated the perimeter and area of each contour, as well as the average value of pixel's intensity and standard deviation (SD) of each image. Independent Student's t-tests were used to compare those variables between PTC and benign nodules, a p-value less than 0.05 was considered significant.

### Network architecture

We retrained two DCNN models in our experiment: VGG-16 and Inception-v3. The architecture of VGG-16 is shown in Table 2; it uses 13 convolutional layers and 3 fully connected layers. The convolutional layers in VGG-16 are all 3×3 convolutional layers with a stride size of 1 and the same padding, and the pooling layers are all 2×2 pooling layers with a stride size of 2. The default input image size of VGG-16 is 224×224. After each pooling layer, the size of the feature map is reduced by half. The last feature map before the fully connected layers is 7×7 with 512 channels and it is expanded into a vector with 25,088 (7×7×512) channels.

**Figure 2:**
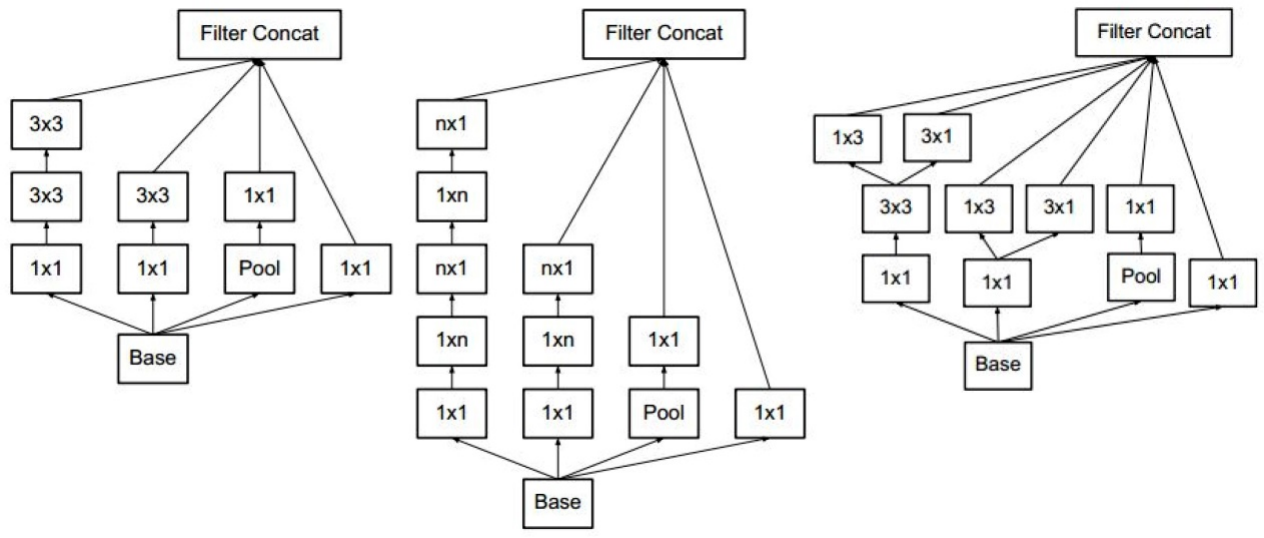
The Inception modules of Inception-v3. The Inception modules of Inception-v3 including Inception module A, B and C from left to right. Each Inception module is composed of several convolutional layers and pooling layers. Pool stands for pooling layer and n×m stands for n×m convolutional layer.

**Figure 3:**
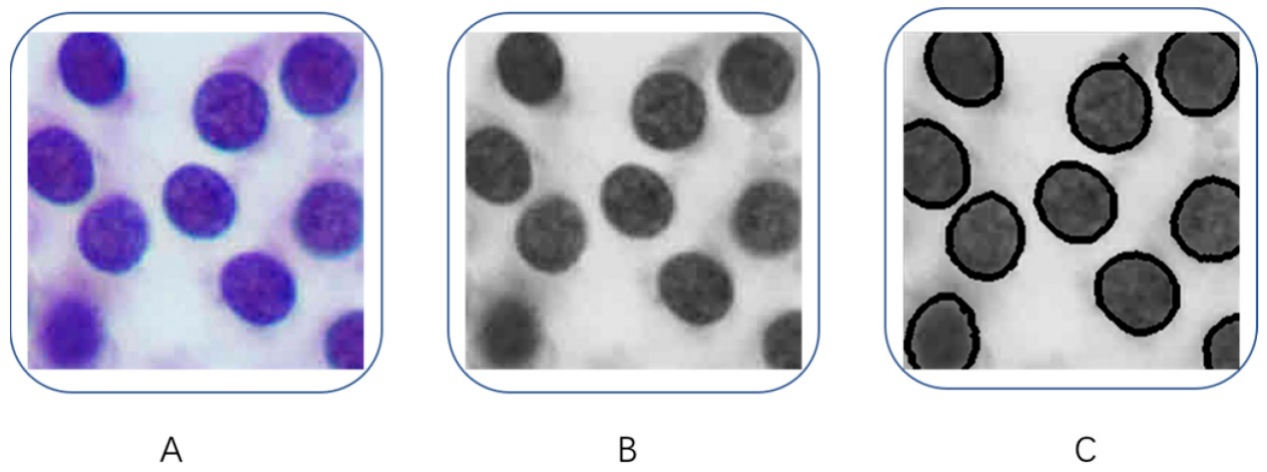
Quantification of tumor cells. (A) Original images, (B) Grayscale images, (C) contours of tumor cells.

The layers of Inception-v3 are shown in Table 3. There were three types of Inception modules in the Inception-v3 model, as shown in Figure 2 (from left to right, Inception modules A, B and C). The Inception modules are well-designed convolution modules that both generate discriminatory features and reduce parameters. Each Inception module is composed of several convolutional and pooling layers in parallel. Small convolutional layers (e.g., 3×3, 1×3, 3×1, and 1×1) are used in the Inception modules to reduce the number of parameters. Inception-v3 stacks 3 Inception A, 5 Inception B and 2 Inception C modules in series. The default input image size for the VGG-16 model is 229×229. After the convolutional and Inception modules, the resulting feature map is 8×8 with 2,048 channels. We used a global pooling layer before the fully connected layer.

The output of the original networks of Inception-v3 and VGG-16 includes 1,000 classes, but our case required only 2 classes, PTC and benign nodules. Therefore, we changed the output channel number of the last layer from 1,000 to 2. We also used a dropout rate of 50% during the training process. The dropout process randomly discards some layer inputs and is used to avoid overfitting.

We used the pretrained models offered by TensorFlow and finetuned them using cytological images. The models were pretrained on the ImageNet dataset and can be found in the TensorFlow-Slim image classification library. We initialized the parameters from the pretrained model because ImageNet had approximately 14,000,000 images, but we had only 279 images. It was difficult to train the models with such a small number of images because deep networks have a large number of parameters. Pretraining can speed up network convergence.

## Results

We used both VGG-16 and Inception-v3 in the experiments. The default image size of VGG-16 was 224×224, which was the same as the sizes of the images in the dataset; however, the default image size of Inception-v3 was 299×299. Consequently, we resized the images to 299×299 when using Inception-v3 to train and test. We trained the two models on the training data and tested them on the test data. Table 4 shows the models' diagnostic efficiency of the test data. Using the VGG-16 model, the DCNN achieved an accuracy of 97.66% on the fragmented images; however, among the three misdiagnosed images, two belonged to one patient; therefore, two patients in the test group received false positively diagnoses by the DCNN. From a patient-wise viewpoint, the accuracy rate was 95% (38/40). Using the Inception-v3 model, the results included nine misdiagnosed fragmented images, among which 5 patients were given a false positively diagnosis. The one false negative diagnosis would not affect the patient's final diagnosis; thus, the total accuracy rate for Inception-v3 was 87.5% (35/40). In these experiments, the VGG-16 results were much better than those of the Inception-v3 model.

The data of quantification were shown in Table 5, firstly, the contours of malignant tumors in fragmented images were more than the benign tumors(61.01±17.10 vs 47.00±24.08, p<0.001), which reflected the fact that PTC tumor cells were more crowded on the cytological images. Secondly, the perimeter (134.99±21.42 vs 62.40±29.15, p<0.001)and area (1770.89±627.22 vs 1157.27±722.23, p=0.013 ) of each PTC cell nucleus were also bigger in the PTC indicating that nuceus of PTC were larger than the benign ones. Last but not least, the means of pixel intensity (165.84±26.33 vs 132.94±28.73, p<0.001) were higher in PTC, which suggested that PTC nuceus have stronger staining.

**Figure 4:**
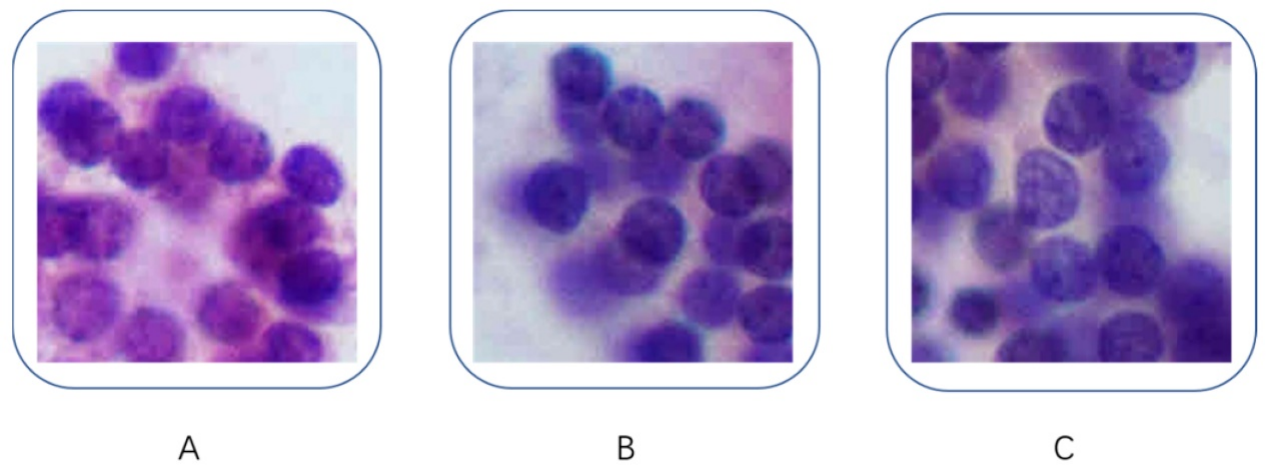
Misdiagnosed fragmented images. (B) and (C) were cropped from same image. A: contour 17, perimeter 149.67, area 1685.91, mean pixel intensity 163.47; B: Contour 19, perimeter 107.17, area 1469.83, mean pixel intensity 165.35; C: Contour 21, perimeter 127.00, area 1839.65, mean pixel intensity 182.79.

After the comparison, we chose VGG-16 over Inception-v3 for further investigation. We further investigated the misdiagnosed images to analyze the reasons for failure. Figure 4 shows the three misdiagnosed fragmented images: note that Figure 4B and Figure 4C were cropped from the same image. The cytopathologists who reviewed these three images considered them to be typical benign nodules, however, the quantification data indicated that those images were more like PTC except the contours (Figure 4A: contour 17, perimeter 149.67, area 1685.91, mean pixel intensity 163.47; Figure 4B, Contour 19, perimeter 107.17, area 1469.83, mean pixel intensity 165.35; Figure 4C, Contour 21, perimeter 127.00, area 1839.65, mean pixel intensity 182.79).

## Discussion

Deep learning has a good performance in image classification, in recent years, a series of deep learning models have been applied to image classification, such as AlexNet, VGGNet and InceptionNet. VGG-16 is a deep convolutional neural network consist of 16 layers that is combined by many 3×3 convolutional layers and 2×2 pooling layers repeatedly, and VGG-16 has a remarkable feature extraction's capability so that it can obtain a good effect in image classification. VGG-16 has a better feature learning ability than AlexNet because it's deeper than AlexNet and it can get more sparse features than AlexNet. Because VGG-16 just uses 3×3 convolution layer and 2×2 pooling layer repeatedly, so that VGG-16 is relatively simpler than InceptionNet, so it has a better generalization's ability, and it can adapt to a variety of data sets including tumor images. As for InceptionNet, there are different sizes of convolution kernels in InceptionNet, so IncetionNet is more suitable for multi-size target's classification, but our tumor images are collected at the same resolution, so InceptionNet is not suitable. In our experiment, VGG-16 achieved an accuracy rate was 95% (38/40) patient wisely, while the accuracy rate was 95% (38/40) for Inception-v3, which confirmed the advantage of VGG-16 in tumor image classification.

FNA cytology is a well-accepted method for diagnosing PTC, and it has an estimated accuracy of approximately 89-95% 20. With the VGG-16 model and selected patients, DCNN achieved 97.66% accuracy in fragmented images and a patient-wise accuracy rate of 95%. Previously, Teramoto A et al developed an automated scheme of DCNN to classify adenocarcinoma, squamous cell carcinoma (SCC), and small cell carcinoma in lung cancer using cytological images, the accuracy rate of classification was 70% 12. Momeni-Boroujeni et al reported a study using multilayer neural network (MNN) to distinguish benign from malignant pancreatic nodules using cytological images, which achieved 100% accuracy, and it can categorize atypical cases into benign or malignant with 77% accuracy 21. Compares to these studies, our results were quite satisfactory, however, there is still room for improvement. It is also worth mentioning that the VGG-16 model did not result in any false negative diagnoses, which indicates that it may be a candidate for use as a screening tool to reduce cytopathologists' workloads. Because computer screening systems have already been introduced to the practice of cervical cytology 22, further study is warranted to validate their application in thyroid or other types of disease.

In this study, we also analyzed misdiagnosed fragmented images, all three images were from benign nodules and were misdiagnosed as PTC. Although the quantification data showed that the perimeter, area and means of pixel intensity of those images were close to PTC, our cytopathologists considered those images to be typical benign nodules. In that case we assumed that the DCNN was making the diagnosis based on the size and staining of the nucleus but not the shape of it, future study should work a way for training the network to differentiate the cellular and nucleus morphology.

The cytological images used in this study all matched the Bethesda classes II, V and VI and included only the PTC pathology subtype. Because it has now been demonstrated that a DCNN can be used to differentiate PTC from benign nodules, future studies should investigate its use in Bethesda class III and IV images, which show atypia of undetermined significance or follicular lesions of undetermined significance, follicular neoplasm or are suspicious for a follicular neoplasm 19. Since this is a pilot study, we have only included a few typical cytological images, in the future study, we could expand the sample size from our hospital or search cooperation with another institution, and there is a chance that we may post our system online so that users can conduct their own assessment.

## Conclusions

In summary, after training with a large dataset, the VGG-16 model of DCNN showed great potential for facilitating the PTC diagnoses using cytological images. The contours, perimeter, area and mean of pixel intensity of PTC in fragmented images were more than the benign nodules.

## Figures and Tables

**Table 1:** Number of fragmented images in the dataset.

Cytological type	Training data	Test data	Total
PTC	407	69	476
Benign nodule	352	59	411
All type	759	128	887

**Table 2:** Architecture of VGG-16 network

Layer	Patch size	Input size
conv×2	3×3/1	3×224×224
pool	2×2	64×224×224
conv×2	3×3/1	64×112×112
pool	2×2	128×112×112
conv×3	3×3/1	128×56×56
pool	2×2	256×56×56
conv×3	3×3/1	256×28×28
pool	2×2	512×28×28
conv×3	3×3/1	512×14×14
pool	2×2	512×14×14
fc	25088×4096	25088
fc	4096×4096	4096
fc	4096×2	4096
softmax	classifier	2

Conv stands for convolutional layer, pool stands for pooling layer and fc stands for fully connected layer. Patch size is the kernel size of convolutional layer, pooling layer or fully connected layer. Input size is feature map input size of the layer.

**Table 3:** Architecture of Inception-v3 network

Layer	Patch size	Input size
conv	3×3/2	229×229×3
conv	3×3/1	149×149×32
conv padded	3×3/1	147×147×32
pool	3×3/2	147×147×64
conv	3×3/1	73×73×64
conv	3×3/2	71×71×80
conv	3×3/1	35×35×192
Inception A×3	------	35×35×288
Inception B×5	------	17×17×768
Inception C×2	------	8×8×1280
pool	8×8	8×8×2048
linear	logits	2048
softmax	classifier	2

Conv stands for convolutional layer, pool stands for pooling layer and fc stands for fully connected layer. Patch size is the kernel size of convolutional layer, pooling layer or fully connected layer. Input size is feature map input size of the layer.

**Table 4:** Diagnostic efficiency of VGG-16 and Inception-v3 on test data (fragmented images)

Model	VGG-16	Inception-v3
Accuracy	97.66%	92.75%
Sensitivity	100%	98.55%
Specificity	94.91%	86.44%
Positive predictive value	95.83%	89.47%
Negative predictive value	100%	98.08%

**Table 5:** Quantification of tumor cells in fragmented images of malignant and benign thyroid tumors.

	Malignant	Benign	p value
Contour	61.01±17.10	47.00±24.08	< 0.001
Perimeter	134.99±21.42	62.40±29.15	< 0.001
Area	1770.89±627.22	1157.27±722.23	0.013
Mean of pixel intensity	165.84±26.33	132.94±28.73	< 0.001

## Abbreviations

DCNN: deep convolutional neural network
PTC: papillary thyroid carcinoma
ATA: American Thyroid Association
FNA: fine-needle aspiration
FTC: follicular thyroid carcinoma
MTC: medullary thyroid carcinoma
ATC: anaplastic thyroid carcinoma
ML: machine learning
SVM: support vector machine
FUSCC: Fudan University Shanghai Cancer Center
LBC: liquid-based cytology
H&E: hematoxylin-eosin
SCC: squamous cell carcinoma
MNN: multilayer neural network
